# Theranostics with somatostatin receptor antagonists in SCLC: Correlation of ^68^Ga-SSO120 PET with immunohistochemistry and survival

**DOI:** 10.7150/thno.98819

**Published:** 2024-08-26

**Authors:** Ilektra Antonia Mavroeidi, Anna Romanowicz, Tristan Haake, Johannes Wienker, Martin Metzenmacher, Kaid Darwiche, Filiz Oezkan, Servet Bölükbas, Martin Stuschke, Lale Umutlu, Marcel Opitz, Michael Nader, Rainer Hamacher, Jens Siveke, Jane Winantea, Wolfgang P. Fendler, Marcel Wiesweg, Wilfried E. E. Eberhardt, Ken Herrmann, Dirk Theegarten, Martin Schuler, Hubertus Hautzel, David Kersting

**Affiliations:** 1 Department of Medical Oncology, West German Cancer Center (WTZ), University Hospital Essen, University of Duisburg-Essen, Essen, Germany.; 2 German Cancer Consortium (DKTK), Partner Site University Hospital Essen, Essen, Germany.; 3 Bridge Institute of Experimental Tumor Therapy (BIT) and Division of Solid Tumor Translational Oncology (DKTK), West German Cancer Center, University Hospital Essen, University of Duisburg-Essen, Essen, Germany.; 4 Department of Nuclear Medicine, West German Cancer Center (WTZ), University Hospital Essen, University of Duisburg-Essen, Essen, Germany; 5 Institute of Pathology, University Hospital Essen, University of Duisburg-Essen, Essen, Germany.; 6 Department of Pulmonary Medicine, Section of Interventional Pulmonology, West German Cancer Center (WTZ), University Medicine Essen - Ruhrlandklinik, University of Duisburg-Essen, Essen, Germany.; 7 Division of Thoracic Oncology, West German Lung Center, University Medicine Essen - Ruhrlandklinik, University of Duisburg-Essen, Essen, Germany.; 8 National Center for Tumor Diseases (NCT) West, Essen, Germany.; 9 Department of Thoracic Surgery and Thoracic Endoscopy, West German Cancer Center (WTZ), University Medicine Essen - Ruhrlandklinik, University of Duisburg-Essen, Essen, Germany.; 10 Department of Radiotherapy, West German Cancer Center (WTZ), University Hospital Essen, University of Duisburg-Essen, Essen, Germany; 11 Institute of Diagnostic, Interventional Radiology and Neuroradiology, West German Cancer Center (WTZ), University Hospital Essen, University of Duisburg-Essen, Essen, Germany.

**Keywords:** ^68^Ga-SSO120, PET, SCLC, SSTR, IHC

## Abstract

**Rationale:** Positron Emission Tomography (PET) using the somatostatin receptor 2 (SSTR2)-antagonist satoreotide trizoxetan (^68^Ga-SSO120) is a novel, promising imaging modality for small-cell lung cancer (SCLC), which holds potential for theranostic applications. This study aims to correlate uptake in PET imaging with SSTR2 expression in immunohistochemistry (IHC) and to assess the prognostic value of ^68^Ga-SSO120 PET at initial staging of patients with SCLC.

**Methods:** We analyzed patients who underwent ^68^Ga-SSO120 PET/CT during initial diagnostic workup of SCLC as part of institutional standard-of-care. SSTR2 expression in IHC was evaluated on a 4-level scale and correlated with normalized standardized uptake values and tumor-to-liver ratios (SUV_max_ and TLR_peak_) in ^68^Ga-SSO120 PET on a lesion level. Highest lesion SUV_max_/TLR_peak_ per patient, SSTR2 score in IHC, M status according to TNM classification, and other parameters were analyzed for association with overall survival (OS) and time to treatment failure (TTF) by univariate, multivariate (cut-off values were identified on data for best separation), and stratified Cox regression.

**Results:** We included 54 patients (24 men/30 women, median age 65 years, 21 M0/33 M1 according to TNM classification). In 43 patients with available surplus tumor tissue samples, hottest lesion SUV_max_/TLR_peak_ showed a significant correlation with the level of SSTR2-expression by tumor cells in IHC (Spearman's rho 0.86/0.81, both p < 0.001; ANOVA p < 0.001). High SSTR2 expression in IHC, ^68^Ga-SSO120 SUV_max_ and TLR_peak_ of the hottest lesion per patient, whole-body TLR_mean_, MTV, TLG, M status, and serum LDH showed a significant association with inferior TTF/OS in univariate analysis. In separate multivariate Cox regression (including sex, age, M stage, and LDH) higher hottest-lesion TLR_peak_ showed a significant association with shorter OS (HR = 0.26, 95%CI: 0.08-0.84, p = 0.02) and SSTR2 expression in IHC with significantly shorter TTF (HR = 0.24, 95%CI: 0.08-0.71, p = 0.001) and OS (HR = 0.22, 95%CI: 0.06-0.84, p = 0.03). In total, 12 patients (22.2%) showed low (< 1), 21 (38.9%) intermediate (≥ 1 but < 2), 14 (25.9%) high (≥ 2 but < 5), and 7 (13.0%) very high (≥ 5) whole-body mean TLR_mean_.

**Conclusion:** In patients with SCLC, SSTR2 expression assessed by ^68^Ga-SSO120 PET and by IHC were closely correlated and associated with shorter survival. More than 75% of patients showed higher whole-body^ 68^Ga-SSO120 tumor uptake than liver uptake and almost 40% high or very high uptake, possibly paving the way towards theranostic applications.

## Introduction

The increasing importance of theranostics, particularly radiotheranostics, in oncology is evident 1. This is driven by the unmet clinical need to understand and face the heterogeneity of response of tumors to standard therapies and by growing interest in potential novel clinical applications, resulting in an increasing number of available target structures 2. In this context, molecular imaging holds tremendous promise to validate target structures. In comparison to conventional techniques like immunohistochemistry (IHC), non-invasive whole-body molecular imaging offers distinct advantages, particularly in capturing the temporal and spatial tumor heterogeneity with greater fidelity 3. In the multidisciplinary management of patients with lung cancer, molecular imaging of glucose metabolism by ^18^F-FDG positron emission tomography (PET) plays a crucial role in treatment decision-making for both small cell lung cancer (SCLC) and non-small cell lung cancer (NSCLC) 4, 5. However, no theranostic applications have been widely established for these most frequent thoracic malignancies yet.

SCLC is a highly aggressive tumor with a dismal prognosis, accounting for approximately 15% of lung cancer diagnoses 6. While large parts of the molecular profile of SCLC remain untargetable, neuroendocrine characteristics with notable expression of type 2 somatostatin receptors (SSTR2) in a relevant fraction of patients 7 suggest a potential for SSTR2-directed theranostics 8. SSTR-directed peptide receptor radionuclide therapy (PRRT) using SSTR-agonists like ^177^Lu-DOTATATE or ^177^Lu-DOTATOC and their ^68^Ga-labeled counterparts for PET imaging have not only been approved in gastroenteropancreatic neuroendocrine tumors (GEP-NETs) 9, 10, but are also successfully applied in lung NETs 11, 12. In patients with SCLC, however, molecular imaging with SSTR2-agonists yielded inconsistent results with high PET tracer accumulation only in selected subgroups of patients 13, and SSTR2-agonist PRRT has not found its way into clinical practice. Here, SSTR2-antagonists, like the theranostic pair ^68^Ga-satoreotide trizoxetan/^177^Lu-satoreotide tetraxetan (^68^Ga-SSO120/^177^Lu-SSO110, previously ^68^Ga-OPS-202/^177^Lu-OPS-201 or ^68^Ga-NODAGA-JR11/^177^Lu-DOTA-JR11), offer higher tumor uptake and prolonged retention times, potentially due to their binding to SSTRs in both active and inactive states 14. Initial clinical applications in GEP-NETs have demonstrated improved sensitivity in PET imaging 15 and higher tumor-absorbed doses in PRRT 14. Moreover, we recently found comparable detection rates of ^68^Ga-SSO120 PET to the gold standard of ^18^F-FDG PET in the initial staging of patients with SCLC and high uptake in up to 40% of patients 16.

This highlights the potential of radiotheranostics using SSTR2-antagonists in this dismal disease. However, in SCLC, lesion uptake in ^68^Ga-SSO120 PET as a biomarker of SSTR2-expression has not been validated against histopathological examination. Furthermore, it remains to be elucidated whether SSTR2-expression in SCLC is associated with favorable prognosis, as previously assumed, or an indicator of specific molecular subtypes and poorer prognosis, as suggested by more recent literature 17.

Therefore, the primary objective of this study is to investigate the correlation between tracer uptake in ^68^Ga-SSO120 PET and expression of SSTR2 in IHC in patients with SCLC. Additionally, the study aims to analyze the prognostic potential of SSTR2-expression assessed by ^68^Ga-SSO120 PET or by IHC for time to treatment failure (TTF) and overall survival (OS) in comparison with established clinical and imaging-based parameters. Lastly, patients are stratified based on their whole-body SSTR2-expression to provide insights into patient eligibility for SSTR2-antagonist PRRT.

## Materials and Methods

### Patients/Ethics

We conducted a retrospective review of our institutional database, identifying patients who underwent ^68^Ga-SSO120 PET/CT as an institutional standard-of-care for staging of SCLC with neuroendocrine differentiation (based on IHC for CD56, synaptophysin SP11, and thyroid transcription factor TTF-1). For further analysis, we specifically selected patients who were tested with ^68^Ga-SSO120 PET in primary diagnostic workup at the initiation of first-line therapy (allowing PET imaging before, within, or after a first cycle of primary chemotherapy). Clinical data were retrieved from the patients' electronic health records system encompassing demographics, clinical history, therapy lines, and blood results (serum lactate dehydrogenase (LDH)). Additionally, survival data were obtained from our institutional Center for Cancer Registry encompassing governmental registration data.

Prior to undergoing clinical PET examinations, patients provided written informed consent. The study received approval from the local institutional ethics committee at the University of Duisburg-Essen, medical faculty, under the ethics protocol number 22-11013-BO. The committee waived the need for study-specific consent.

### PET/CT imaging

PET/CT images were acquired on a Biograph Vision 600, a Biograph mCT (both Siemens Healthineers), or a Vereos (Philips Healthcare) PET/CT system 64 ± 16 min (mean ± standard deviation SD) after administration of 141.8 ± 29.0 MBq (mean ± SD) of ^68^Ga-SSO120. PET/CT acquisition started with a contrast enhanced whole-body CT; the CT images were used for attenuation correction and anatomical localization of PET uptake. If a contrast enhanced whole-body CT was already clinically available within 4 weeks prior to the examination date, a low-dose CT was performed instead. PET/CT acquisition and image reconstruction was performed according to our established institutional protocols for ^68^Ga-based PET tracers 18.

Where available within a two-week interval before or after ^68^Ga-SSO120 PET, additional staging ^18^F-FDG PET/CT was considered for comparison under the condition that no significant morphological differences were observed in the CT images (stable disease according to RECIST 1.1 criteria).

### PET image analysis

Analysis of PET images was independently performed by three nuclear medicine physicians with several years of experience in PET reporting (A.R., D.K., and H.H.). In case of discrepant findings, re-evaluation for consensus decision making was performed. Segmentation of PET-positive tumor was performed using the Syngo.via software solution (Siemens Medical Solutions, Erlangen, Germany) in a semi-automatic approach. First, a spherical volume-of-interest (VOI) in the right liver lobe (in analogy to Positron Emission Response Criteria in Solid Tumors (PERCIST) 1.0 criteria) was automatically determined to estimate the standardized uptake values SUV_max_, SUV_peak_, and SUV_mean_ 19. Next, all foci with a SUV_max_ value ≥ (1.5 x SUV_mean_ + 2 x SD of SUV_mean_ in the liver VOI) were automatically segmented, determining lesion boundaries by a 41-% local SUV_max_ threshold derived from current recommendations of the European Association of Nuclear Medicine 20. Lesions with a volume <0.1 mL were not considered. Finally, all segmented foci were manually validated to exclude regions of physiological uptake, and additional lesions were added if visually detected. Here, tumor lesions were defined as regions with focal markedly increased ^68^Ga-SSO120-/^18^F-FDG-uptake compared to local background without physiological explanation. Volume, SUV_max_, SUV_peak_, and SUV_mean_ of the individual tumor VOIs were determined. As a robust measure of lesion tracer uptake, the normalized tumor-to-liver ratios (TLR_peak_ and TLR_mean_) were defined using the liver VOI as reference 21:



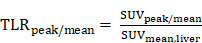



Whole-body SSTR2-expressing tumor volume (SSTR-TV) and metabolic tumor volume (MTV) were defined as the sum of the volumes of all segmented lesions in ^68^Ga-SSO120 and ^18^F-FDG PET, respectively. Whole-body tumor SUV_mean_ and TLR_mean_ were calculated from the SUV_mean_ and TLR_mean_ of all segmented lesions in ^68^Ga-SSO120 and ^18^F-FDG PET per patient, respectively. Total lesion SSTR2-expression (TL-SSTR) and total lesion glycolysis (TLG) were defined as:



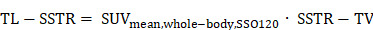



and

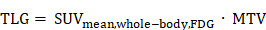



in ^68^Ga-SSO120 PET and ^18^F-FDG PET/CT, respectively. Moreover, the lesion with the highest SUV_max_ and TLR_peak_ value (hottest lesion) and the number of detected lesions per patient in ^68^Ga-SSO120 PET were determined.

### Immunohistochemistry

Systematic endobronchial ultrasound-guided transbronchial needle aspiration (EBUS-TBNA) is routinely performed for mediastinal and hilar lymph node staging and primary tumor diagnostics at our institution leading to a high number of available samples. For eligible patients with available biopsy specimens, histopathologic analysis was conducted. Staining of biopsy specimens was performed with standard hematoxylin and eosin and SSTR2 IHC stains. The biopsy specimens were cut into up to 2 µm thin slices of the bronchoscopic samples and stained by IHC using a polyclonal antibody for SSTR2 (dilution 1:50, incubation time at 36 °C for 40 min, Zytomed Systems, Berlin, Germany). The automated system used for staining was the Ventana Benchmark Ultra in combination with an Optiview DAB IHC detection kit for visualization (Roche, Basel, Switzerland). The staining results were visually evaluated by two experienced pathologists (D.T. and T.H.) who were blinded to the imaging findings. The scoring system used for SSTR2 staining results employed a 4-level scale (SSTR2 score 0: negative, 1: 1-29%, 2: 30-69%, 3: ≥70%), providing a systematic assessment of SSTR2 expression. The SSTR2 score of the examined IHC specimens was correlated with SUV_max_/TLR_peak_ in ^68^Ga-SSO120 PET on a lesion level. For this purpose, the lesion which was biopsied was specifically selected in the PET images to determine its uptake parameters (only in patients with at least one day difference between ^68^Ga-SSO120 PET and ^18^F-FDG PET to avoid uptake interferences when performed on the same day).

### Study endpoints

We defined parameters indicative of tumor load (M status, number of lesions in ^68^Ga-SSO120 PET, LDH), of SSTR2 expression (SSTR2 score in IHC, SUV_max_/TLR_peak_/whole-body tumor SUV_mean_/whole-body tumor TLR_mean_ in ^68^Ga-SSO120 PET), and combination parameters (SSTR-TV, TL-SSTR2). Moreover, two established parameters for survival prediction from ^18^F-FDG PET (MTV and TLG 22) were used for validation.

Primary study endpoints were correlation of SSTR2 expression assessed by IHC with SUV_max_/TLR_peak_ in ^68^Ga-SSO120 PET on a lesion-level and correlation of SSTR2 expression (assessed by IHC and ^68^Ga-SSO120 PET) with TTF and OS. Secondary endpoints included correlation of parameters of tumor load, combination parameters, and MTV/TLG with OS and TTF, comparison of whole-body tumor SUV_mean_ and SSTR-TV in ^68^Ga-SSO120 PET with SUV_mean_ and MTV in ^18^F-FDG PET/CT, as well as assessment of patient-based mean ^68^Ga-SSO120 uptake (assessed by mean TLR_peak_ per patient) as surrogate of applicability of theranostic approaches.

TTF was defined as the time from ^68^Ga-SSO120 PET until the initiation of a second line therapy after documented disease progression or death. For patients without documented progression, TTF was censored on the date the patient was last known to be non-progressing after first-line therapy. OS was defined as the time from ^68^Ga-SSO120 PET to the date of death; patients without documented death on the cut-off date were censored on the date the patient was last known to be alive. For imaging studies, different definitions of the starting date for calculation of OS and TTF are frequently used. As in this study the time from ^68^Ga-SSO120 PET aligns with the time of recruitment and differs from the initiation of chemotherapy by a maximum several days, it is a precise and accurate point of reference.

### Statistical analysis/software

For comparison of non-normally distributed data, a non-parametric Mann-Whitney U test was employed, with measures reported as median and interquartile range (QR). Beforehand, data was assessed for parametric distribution using the Shapiro-Wilk test. The correlation of SSTR2 expression in IHC and uptake in ^68^Ga-SSO120 PET was evaluated using ANOVA analysis and the Spearman rank correlation coefficient; linear regression after semi-logarithmic transformation was performed to assess for exponential relationship.

To determine the association of PET data, IHC SSTR2 score, LDH, and M status with survival data, uni- and multivariate Cox regression analyses were performed; median follow up was calculated by the reverse Kaplan-Meier method. Continuous variables were binarized using cut-off values that were identified on data for optimal separation and Hazard ratios (HR) with 95%-confidence intervals were calculated. For parameters of SSTR expression (primary endpoint), additional Cox regression analyses of continuous variables were calculated. The results of survival analyses are visually presented using Kaplan-Meier curves. Stratified Cox regression analyses were performed to account for different baseline hazards between patients with M0 or M1 status. Adjusted Kaplan Meier curves were used to adapt survival for confounding parameters of tumor load and clinical characteristics.

In all statistical tests, p-values (p) <0.05 were regarded significant. All statistical evaluations were performed using R statistical software in version 4.3.2 (R Foundation for Statistical Computing, Vienna, Austria, www.R-project.org). Cut-off values for binarization in Cox regression analyses were calculated using the surv_cutpoint function from the survminer package; adjusted Kaplan Meier curves were generated using the adjustedCurves package based on the direct standardization method 23. The graphical abstract was created using BioRender.com (BioRender, San Francisco, USA, www.BioRender.com).

### Data availability statement

The data generated in this study are available upon request from the corresponding author.

## Results

### Patient Characteristics

**Figure 1:**
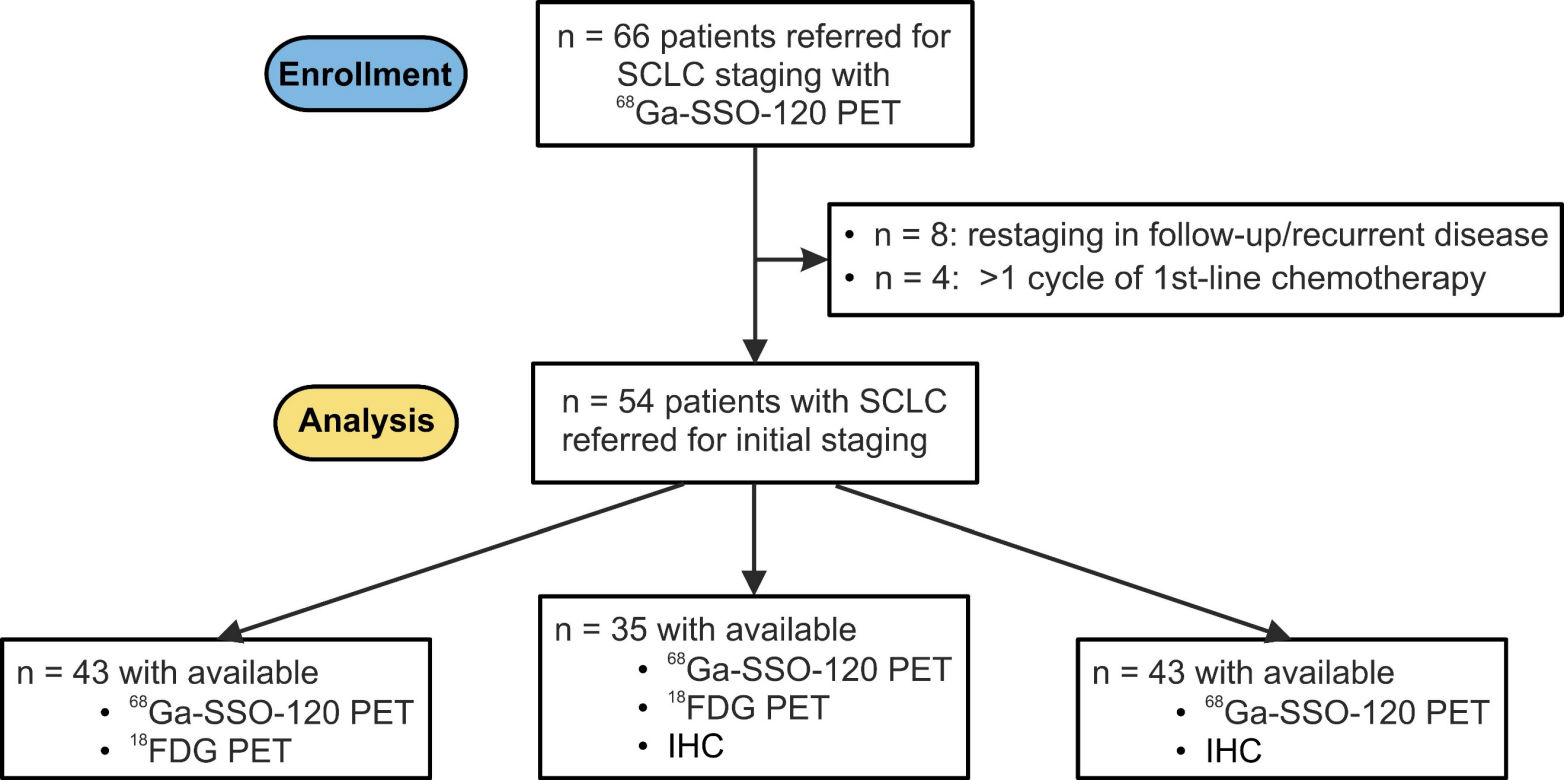
** Patient Flow Chart.** Consort flow diagram showing patients with SCLC who underwent ^68^Ga-SSO120 PET at our institution (University Hospital Essen) between May 2022 and November 2023 and patients who were analyzed according to the inclusion criteria. IHC: immunohistochemistry.

Between May 2022 and November 2023, a total of 66 patients underwent ^68^Ga-SSO120 PET/CT for staging or restaging of SCLC at our institution (University Hospital Essen). Of these, 54 patients underwent ^68^Ga-SSO120 PET/CT at initial staging and were included in this analysis. Database closure for survival status was end of January 2024. Evaluable additional staging ^18^F-FDG PET/CT was available for 43 patients (75.4%). **Figure 1** shows a consort diagram depicting patient inclusion and study workflow.

In the study cohort, both sexes were equally represented (24 men, 30 women) and median age was 65 years. According to the current TNM classification (World Health Organization WHO/International Association for the Study of Lung Cancer IALSC 8^th^ edition 24), 7 patients were classified as stage IIIA (12.9%), 6 as IIIB (11.1%), 8 as IIIC (14.8%), and 33 as IV (61.1%); 21 patients without distant metastases (M0) showed limited disease (38.1%) and 33 with distant metastases (M1) extensive disease (61.1%) according to the Veterans Administration Lung Study Group (VALG) classification. Detailed patient characteristics are given in **Supplemental Table S1.**

In first line, 21 patients received platinum-based chemotherapy (platinum/etoposid) and 33 patients a platinum-based chemo-immunotherapy combination (25 cisplatin/etoposid/durvalumab, 8 carboplatin/etoposid/atezolizumab) according to current guidelines 25. The median number of first-line therapy cycles was 4. Median TTF was 9.5 mos (interquartile range QR: 7.3-14.9 mos) and median OS was 16.1 mos (QR: 13.0 mos - not estimable). Only 2 patients progressed during the first line therapy. However, at censoring point, 29 patients had progressed and 21 patients were deceased; 4 patients were lost to follow-up for OS. Median LDH was 263 U/l (QR: 217-345).

### PET Imaging Results

All included patients had at least one SSTR2-positive lesion in ^68^Ga-SSO120 PET with a median number of 7 detected lesions per patient (QR: 2-18). Median SUV_max_ and TLR_peak_ of the hottest lesion per patient were 13.1 (QR: 5.9-27.6) and 2.9 (QR: 1.4-8.1), respectively. Median SSTR-TV, TL-SSTR, whole-body tumor SUV_mean_, and whole-body tumor TLR_mean_ were 96.7 mL (QR: 33.6-174.8 mL), 343.1 (QR: 128.5-827.0), 3.8 (QR: 2.7-11.5), and 1.5 (1.0-4.1), respectively. Median MTV was 103.3 mL (QR: 42.6-240.2 mL) and median TLG was 800.0 (QR: 253.0-1760.0).

When comparing patients with M0 versus M1 status, the number of lesions was significantly higher in patients with M1 status. Also, tumor volumes (SSTR-TV and MTV) and TLG were higher in M1, almost reaching statistical significance. Parameters of SSTR2 expression and LDH were not significantly different between both groups. Detailed results are given in **Table 1**.

A comparison of SSTR2-TV versus MTV and whole-body tumor SUV_mean_ from ^68^Ga-SSO120 PET versus ^18^F-FDG PET revealed no correlation (scatter plots are presented in **Supplemental Figure S1**).

### Correlation of ^68^Ga-SSO120 PET with SSTR2 expression in IHC

**Figure 2:**
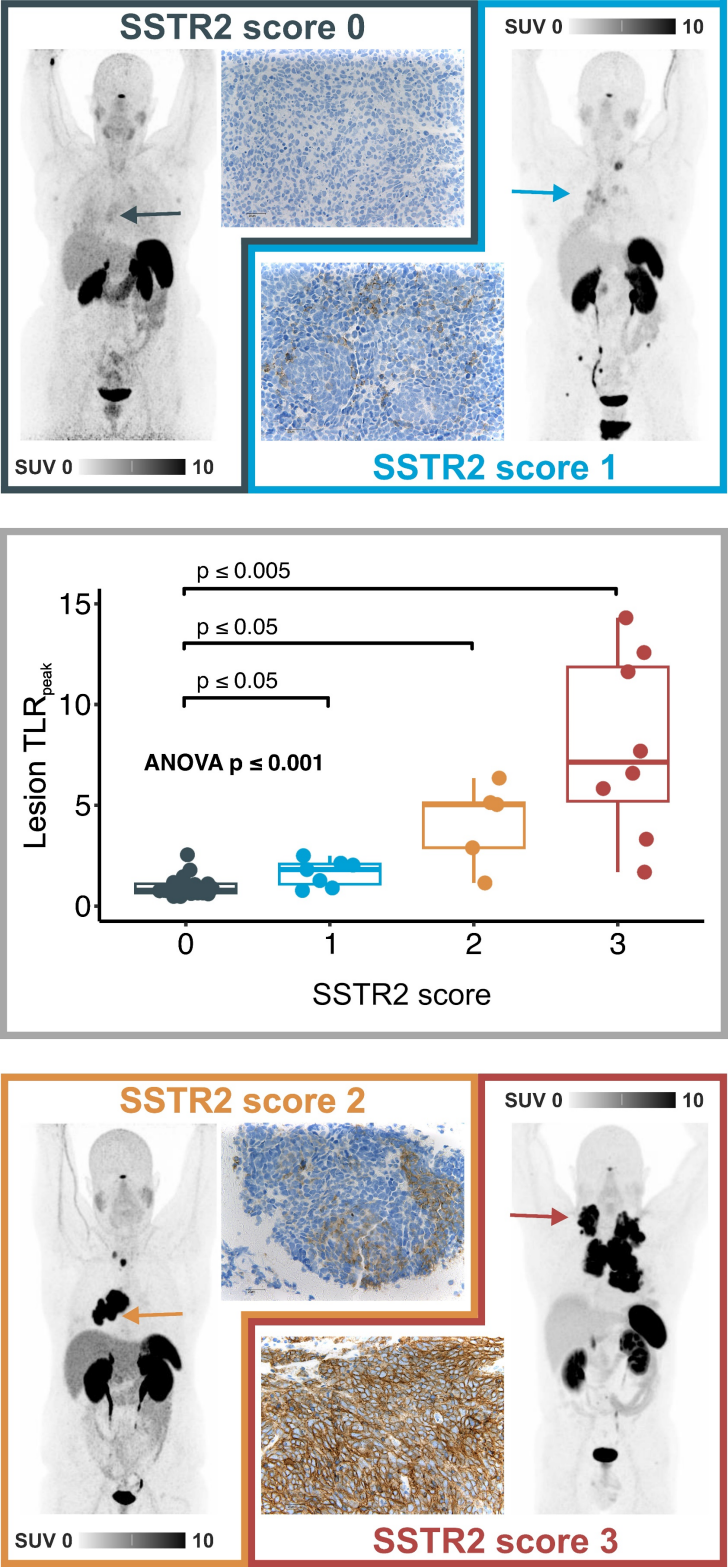
** Correlation of ^68^Ga-SSO120 uptake and IHC patterns.** Top/Bottom: ^68^Ga-SSO120 PET (maximum-intensity projection) and IHC image examples of patients with low uptake and IHC score of 0 (lesion TLR_peak_: 1.2, whole-body tumor TLR_mean_: 0.83), intermediate uptake and IHC score of 1 (lesion TLR_peak_: 1.8, whole-body tumor TLR_mean_: 1.6), high uptake and IHC score of 2 (lesion TLR_peak_: 5.1, whole-body tumor TLR_mean_: 4.8), and very high ^68^Ga-SSO120 uptake und IHC SSTR2 score of 3 (lesion TLR_peak_: 11.6, whole-body tumor TLR_mean_: 8.5). Arrows indicate the lesion that was evaluated in IHC in the PET images. Middle: Box-plot representation of lesion-based ^68^Ga-SSO120 TLR_peak_ for different SSTR2 IHC score groups. Correlation was tested with ANOVA, p-values between individual groups refer to results from Mann-Whitney U test. Horizontal line: median, hinges: first and third quartiles, whiskers: lowest/highest within 1.5 * inter-quartile range of the hinge.

A total of 43 specimens were available for IHC analysis. 21 patients (48.8%) were negative for SSTR2 expression in IHC (SSTR2 score 0), 8 patients (18.6%) were evaluated with low (SSTR2 score 1), 5 (11.6%) with intermediate (SSTR2 score 2), and 9 (20.9%) with high expression (SSTR2 score 3). In ANOVA analysis, SUV_max_ and TLR_peak_ values were significantly higher in lesions with higher SSTR2 score (p < 0.001) and a strong monotonical correlation was found between SSTR2 score in IHC and corresponding lesion SUV_max_ (Spearman's rho 0.86, p < 0.001) and TLR_peak_ (Spearman's rho 0.81, p < 0.001) in ^68^Ga-SSO120 PET. Image examples and a boxplot representation of TLR_peak_ values in patients with different SSTR2 score in IHC are shown in **Figure 2**. In-depth analysis indicated an exponential relationship between SUV_max_/TLR_peak_ and SSTR2 score in IHC (R^2^ for log transformed SUV_max_ /TLR_peak_ in linear regression: 0.76/0.70, details in **Supplemental Figure S2**).

### Univariate survival analyses

Regarding the primary study endpoint, SSTR2 expression both in PET imaging and in IHC analysis was associated with shorter TTF and OS. Statistical significance was reached for SSTR2 score in IHC as well as for SUV_max_ and TLR_peak_ of the hottest lesion per patient and whole-body tumor TLR_mean_ in ^68^Ga-SSO120 PET. For example, a SSTR2 score ≥1 in IHC was associated with worse TTF (HR = 0.24, 95%CI: 0.09-0.64, p = 0.004) and OS (HR = 0.26, 95%CI 0.07-0.83, p = 0.023). The same holds for a high TLR_peak_ (TTF: HR = 0.44 for TLR_peak_ ≤ 2.9, 95% CI: 0.19-0.96, p = 0.038; OS: HR = 0.23, 95%CI: 0.07-0.71, p = 0.011). This trend was confirmed in analyses of continuous variables (**Supplemental Table S2**).

**Figure 3:**
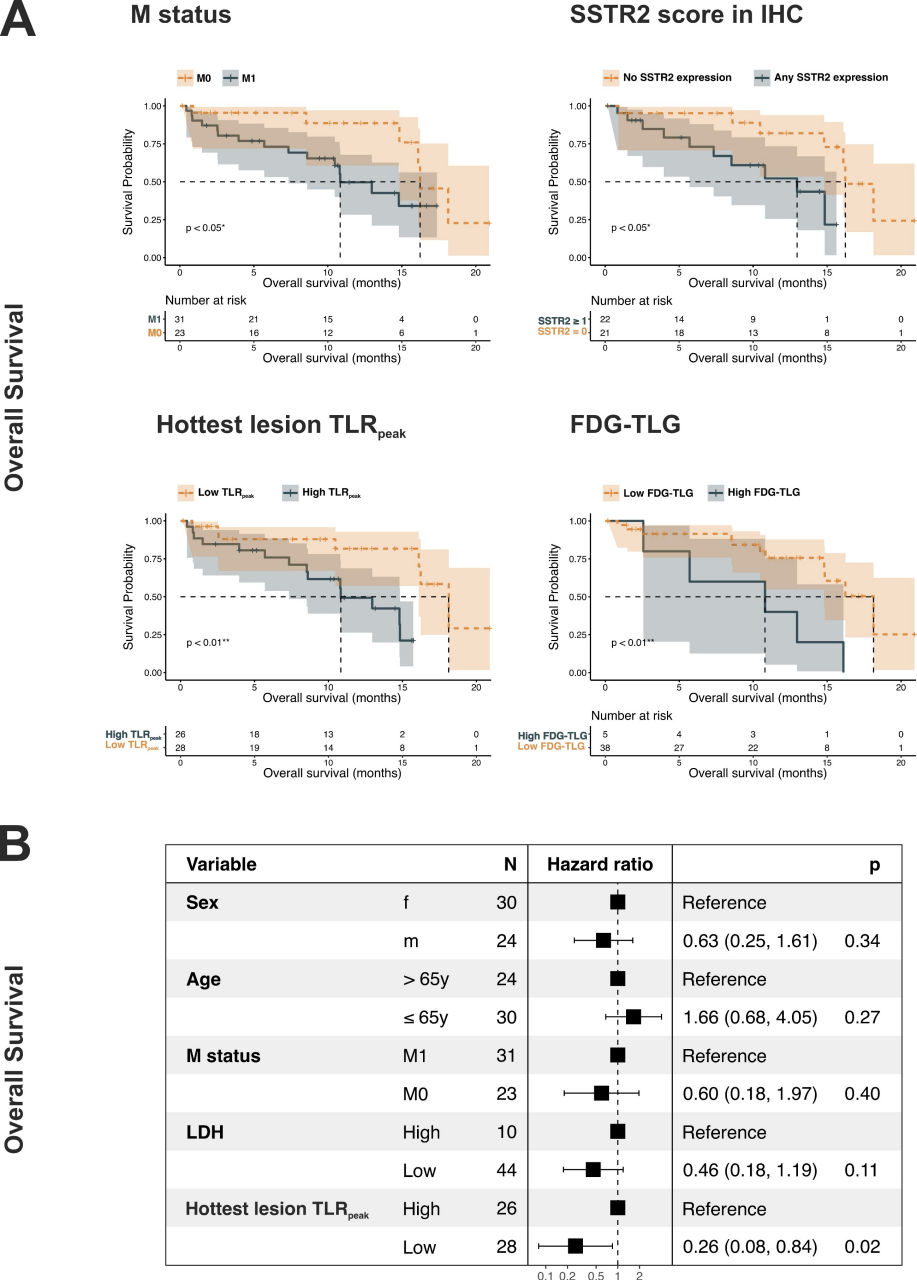
** Survival analyses for OS.** A: Kaplan-Meier curves illustrating OS in correlation to M status, SSTR2 score in IHC, hottest lesion TLR_peak_, and FDG-TLG. B: Forest plot showing the results of the multivariate Cox regression for OS (sex, age (stratified by median), M status, LDH, hottest lesion TLR_peak_). (High LDH: >418 U/l, High hottest lesion TLRpeak: >2.9).

Regarding secondary endpoints (analysis of non-SSTR2 co-variables), parameters indicating higher tumor load (M1 status, higher LDH, and higher number of lesions), combination parameters (higher SSTR-TV and higher TL-SSTR), and higher MTV/TLG showed an association with poorer survival, both in terms of TTF and OS. Here, statistical significance was reached for M status, LDH, MTV, and TLG. Patients with metastasized disease showed a significantly shorter TTF (HR = 0.31, 95%CI: 0.12-0.74, p = 0.004) and OS (HR = 0.34, 95%CI: 0.12-0.95, p = 0.04), while patients with higher TLG exhibited a significantly shorter OS (HR = 0.25, 95%CI: 0.08-0.75, p = 0.01). Details showing median TTF and OS for all parameters are given in **Table 2**. Kaplan-Meier curves for M status, SSTR2 score in IHC, TLR_peak_ of the hottest lesion, and TLG are shown in **Figure 3A** and **Supplemental Figure S3A**.

### Stratified and multivariate survival analyses

To account for different baseline hazards, stratified Cox regression analysis for M status was performed for all parameters that showed significant associations in univariate analyses (SSTR2 score, SUV_max_ and TLR_peak_ of the hottest lesion, whole-body tumor TLR_mean_, LDH, MTV, and TLG). In the stratified analysis, TLR_peak_ of the hottest lesion and TLG yet showed a significant association with shorter OS (TLR_peak_: HR = 0.30, 95%CI: 0.09-0.97, p = 0.0431; TLG: HR = 0.29, 95%CI: 0.09-0.92, p = 0.034), while SSTR2 expression in IHC yet showed a significant association with shorter TTF (HR = 0.32, 95%CI: 0.12-0.87, p = 0.024). Detailed results are shown in **Supplemental Table S3.**

SSTR2 score and TLR_peak_ of the hottest lesion, the IHC- and ^68^Ga-SSO120 PET-derived parameters of SSTR2-expression that showed best significant association with survival, were analyzed in separate multivariate Cox regression with demography- and tumor burden-associated co-variables (sex, age larger/smaller than median, M stage, and LDH). Higher SSTR2 score in IHC and higher TLR_peak_ of the hottest lesion still showed a significant association with shorter survival. In detail, higher TLR_peak_ was associated with significantly shorter OS (HR = 0.26, 95%CI: 0.08-0.84, p = 0.02), and SSTR2 expression in IHC with significantly shorter TTF (HR = 0.24, 95%CI: 0.08-0.71, p = 0.001) and OS (HR = 0.22, 95%CI: 0.06-0.84, p = 0.03). Forest plots providing detailed results are shown in **Figure 3B** and **Supplemental Figures S3B and S4.** Adjusted Kaplan Meier plots (adjusted for the same co-variates) are shown in **Supplemental Figures S5A and S5B.** In these visualizations, SSTR2 expression showed a correlation with poorer survival: Patients with higher TLR_peak_ of the hottest lesion exhibited a shorter adjusted median OS (13.1 mos versus 18.1 mos, 95%CI: 8.5-16.3 mos versus 14.8 mos - not estimable) and TTF (7.9 mos versus 14.8 mos, 95%CI: 6.2-10.8 mos versus 8.1-16.5 mos).

### Analysis of mean tumor SSTR2 expression

**Figure 4:**
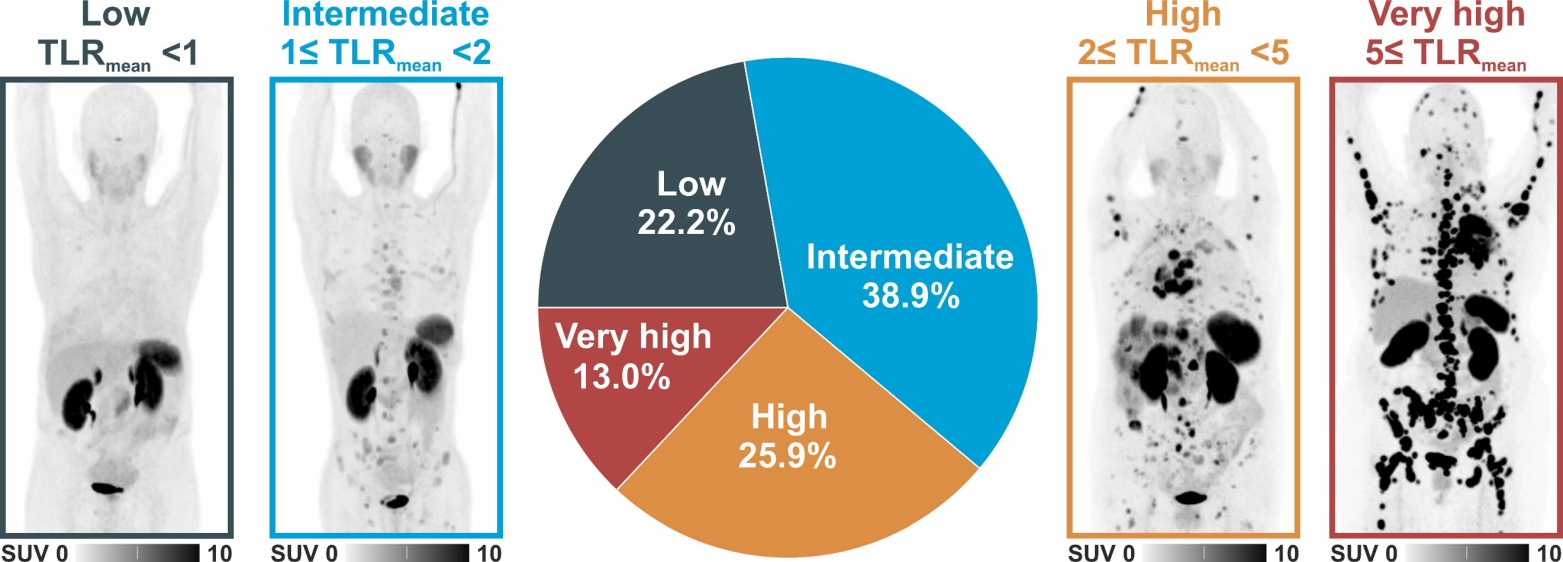
** Lesion-based comparison of ^68^Ga-SSO120.** Distribution of ^68^Ga-SSO120 uptake patterns and image examples (maximum-intensity projections) of patients with low, intermediate, high, very high uptake in^ 68^Ga-SSO120 PET (assessed by whole-body tumor TLR_mean_). Definitions of TLR_mean_ thresholds to define uptake groups are presented in the figure.

Clustering patients according to their whole-body tumor TLR_mean_ as a surrogate parameter to identify possible candidates for radioligand therapy revealed 12 patients (22.2%) with low mean TLR_mean_ (<1), 21 patients (38.9%) with intermediate mean TLR_mean_ (≥1 but <2), 14 patients (25.9%) with high mean TLR_mean_ (≥2 but <5), and 7 patients (13.0%) with very high mean TLR_mean_ (≥5). In this classification 42 patients (77.8%) with intermediate, high, and very high uptake exhibited higher mean uptake than liver uptake and could, therefore, be potential candidates for PRRT. **Figure 4** shows image examples of patients with different uptake levels as assessed by ^68^Ga-SSO120 PET and the distribution of different uptake groups.

## Discussion

This study endeavors to comprehensively investigate the potential of ^68^Ga-SSO120 PET as a biomarker in order to offer an innovative radiotheranostic approach in patients with SCLC. It is the largest study so far to describe the use of SSTR-antagonist PET in SCLC. Primary aim of the study was to enhance the understanding of SSTR2 expression in SCLC in correlation with histopathology, standard of care molecular imaging, and patient outcomes, ultimately contributing to improved personalized management strategies for SCLC patients.

Regarding the first primary study endpoint, a significant correlation between SSTR2 expression in IHC and uptake values in ^68^Ga-SSO120 PET was shown (**Figure 2** and **Supplemental Figure S2**). This validates that ^68^Ga-SSO120 PET reliably visualizes SSTR2 expression in SCLC, so that PET imaging can be used to assess whole-body target expression and select patients in a theranostic setting. PET-guided biopsy could reduce sampling errors and overcome limitations of temporal and spatial tumor heterogeneity. In addition, it could pave the way for theranostic approaches in both metastasized and non-metastasized disease stages of SCLC.

Regarding the second primary study endpoint, SSTR2 expression, as detected by both ^68^Ga-SSO120 PET and IHC, was significantly correlated with shorter TTF and OS (**Figure 3** and **Supplemental Figure S3**), suggesting its potential as a prognostic marker in SCLC. This result could be influenced by differences in SSTR2 expression between patients with M0 and M1 status as well as different baseline hazards. To account for these factors, we conducted various analyses: Firstly, parameters of SSTR2 expression were not significantly different between these two groups (**Table 1**). Secondly, the effect of poorer survival in patients with high SSTR2 expression was also evident in Cox regression stratified for M status (**Supplemental Table S3**), in multivariate Cox regression (**Figure 3B, Supplemental Figure S3B, Supplemental Figure S4**), and in adjusted Kaplan Meier curves (**Supplemental Figure S5**). RNA sequencing results from other recent studies, for example by Lehman et al. 17, also suggest that high SSTR2 expression correlates with unfavorable outcomes in non-metastasized SCLC, emphasizing a role of SSTR2 signaling in progression and survival of tumor cells. This could be a sign of SSTR2-expression in SCLC being indicative of immune evasion and increased tumor cell invasiveness 26, contradicting earlier assumptions derived from NETs, which assumed that SSTR2-expression could indicate less aggressive tumors and a potential for favoring apoptosis 8. This trend is underlined by recent literature stating that absence of SSTR2 expression could activate apoptosis through alternate pathways 26. These observations may hint at different roles of SSTR2-related molecular pathways in different cancer types 26.

There is a very limited, increasing though, number of studies, which tried to explore the prognostic role of SSTR expression both in SCLC and other tumor entities. In SCLC, Sen *et al.* 27 and Lapa *et al.* 13 did not find any statistic significant correlation between SSTR expression and survival. On the other hand, comparable studies in nasopharyngeal carcinomas, gliomas, and thymic carcinomas suggested a negative correlation 28, 29, in alignment with our study.

With regards to the secondary study endpoints (influence of non-SSTR2 co-variables), M status, LDH, as well as MTV and TLG derived from ^18^F-FDG PET were also significantly associated with poorer TTF and OS (**Table 1**, **Supplemental Figure S3A** and** Figure 3**). This is in line with previous results for these markers of tumor burden and metabolic activity 22. LDH and TLG were also significant prognosticators in multivariate or stratified analyses.

About 40% of patients exhibited high or very high ^68^Ga-SSO120 uptake as assessed by their whole-body tumor TLR_mean_ (**Figure 4**). This validates the findings of a preliminary study in a smaller patient subcohort of this study conducted by our group 16, wherein we additionally showcased comparable detection rates between ^68^Ga-SSO120 and ^18^F-FDG PET. This underscores the effectiveness of both imaging modalities for the initial staging of SCLC.

In the realm of personalized medicine and theranostic options, understanding the molecular underpinnings of SCLC tumor biology is crucial for selecting the optimal therapy. In this context, the high uptake in a relevant fraction of patients shows the potential of SSTR-targeted therapies in this patient group. Possible SSTR-targeting therapeutic options comprise (long-acting) somatostatin analogues like lanreotide which are successfully applied in GEP-NETs and pulmonary NETs 30 and targeted radionuclide therapy 31. Nevertheless, in SCLC patients only few applications of somatostatin analogues 32-34 or SSTR-agonist PRRT 13, 35-37 were described and remained without sufficient results for wide clinical applications. For example, Sen *et al.* reported on a total of 67 patients with advanced SCLC who were screened with ^68^Ga-DOTATATE PET/CT. About 50% showed mainly SSTR-positive lesions, however, in contrast to the evaluation at primary staging in our study, an association with survival outcome was not demonstrated. PRRT was performed in 14 patients, resulting in disease control in 5/13 (about 40%) of patients 27. Kim *et al.* enrolled 6 patients with extensive SCLC for a combination therapy of ^177^Lu-DOTATATE with nivolumab; one patient showed a partial response, indicating a favorable efficacy profile and antitumor activity 38.

These data indicate the potential of SSTR-targeted PRRT in patients with SCLC. Moreover, addition of ^177^Lu-DOTATATE to first line chemoimmunotherapy in patients with extensive-disease SCLC in a multi-modal treatment concept is currently investigated in a phase 1 trial (CAAA601A42101, ClinicalTrials registration NCT05142696). Furthermore, a phase 1 trial analyzes RYZ101 (^225^Ac-DOTATATE), an alpha-emitting radiopharmaceutical 39, in a comparable setting (ClinicalTrials registration NCT05595460).

On the other hand, radiolabeled SSTR2-antagonists exhibit improved pharmacokinetic properties and can offer promising novel therapeutic options 14, bearing the potential of improved response rates because of increased tumor uptake and longer residence times 40. For NET patients, tumor doses for ^177^Lu-SSO110 were increased by a factor of up to ten compared to ^177^Lu-DOTATATE 14. Therefore, in SCLC patients with sufficient uptake in PET imaging, SSTR2-antagonist PRRT might be considered in a multi-modal theranostic approach. Notably, mean tumor ^68^Ga-SSO120 uptake was larger than liver uptake in almost 80% of patients (**Figure 4**), which is a typical criterion to evaluate eligibility of patients for radionuclide therapies (Krenning score 41).This indicates that SSTR-antagonist theranostics could open up a promising novel therapeutic option for maintenance or consolidation in patients with both M0 and M1 SCLC, particularly intriguing as higher expression of the target was associated with poorer OS and faster progression.

Until now, no study results of SSTR-antagonist PRRT in SCLC have been described. An ongoing phase Ib study investigates addition of ^177^Lu-SSO110 to maintenance therapy in extensive stage SCLC (protocol presented at European Association of Nuclear Medicine annual meeting 2023). In patients with NETs, a prospective phase I and a prospective phase I/II trial investigated 177-Lu-SSO110 and showed promising clinical efficacy 42, 43. Moreover, the PROMENADE trial compared ^177^Lu-SSO110 treatment with the more established ^177^Lu-DOTATOC therapy in the same patients with progressive standard-therapy refractory meningioma showing a favorable therapeutic index with high disease control rate 44.

Collectively, these findings support that ^68^Ga-SSO120 PET holds promise for the characterization and prognostication of SCLC and could possibly open novel theranostic opportunities. However, special attention might have to be taken on strategies to mitigate adverse effects. For example, in the phase I trial in NET patients, after the 2nd cycle of ^177^Lu-SSO110 therapy, grade 4 hematologic toxicity occurred in four of seven (57%) patients. After adjustment of dose and treatment intervals restricting the cumulative absorbed bone marrow dose to 1 Gy possible hematologic toxicity was resolved 43. This indicates that SSTR-antagonist PRRT can be possible under careful surveillance and individual selection of treatment dosage.

Future research might focus on the evaluation of patients in later therapy lines to validate the target expression against the background of possible clonal evolution and heterogenous uptake patterns. In future, molecular imaging with various tracers might be performed in individual patients to select the optimal theranostic targets. SSTR-antagonists belong to the substances with highest potential due to high expression levels in a large number of patients.

Main limitation of the study is the yet limited number of included patients, although it is a large cohort for this tumor entity. Therefore, multivariate survival analyses in comparison to ^18^F-FDG PET-derived parameters were not performed. Moreover, not all parameters of SSTR-expression were statistically significant predictors of OS and TTF in all analyses. However, the prognostic significance of established parameters like M status and TLG/MTV (the latter in univariate analysis) indicate the validity of the obtained results. Future (prospective) studies with longer follow-up are warranted to investigate SSTR2-antagonist molecular imaging and targeted therapy in patients with SCLC.

## Conclusion

In patients with SCLC, SSTR2 expression assessed by ^68^Ga-SSO120 PET and by IHC were closely correlated and associated with shorter survival. More than 75% of patients showed higher whole-body^ 68^Ga-SSO120 tumor uptake than liver uptake and almost 40% high or very high uptake, possibly paving the way towards theranostic applications.

## Supplementary Material

Supplementary figures and tables.

## Figures and Tables

**Table 1:** M0 versus M1 status

M status	M0	M1	*p*_(Mann-Whitney *U*)_
Number of lesions	4 (2-7.5)	10 (2.5-41.5)	<0.05*
LDH (U/l)	267 (222-306)	262 (241.5-482.5)	0.73
SSTR2 score in IHC	1 (1-2)	2 (1-4)	0.08
SUV_max_ hottest lesion	8.9 (5.8-22.4)	13.6 (6.4-31.6)	0.24
TLR_peak_ hottest lesion	2.3 (1.4-5.3)	3.6 (2.0-10.0)	0.12
Whole-body SUV_mean_	4.9 (3.3-10.3)	3.5 (2.5-11.7)	0.50
Whole-body TLR_mean_	1.6 (1.0-3.4)	1.5 (1.0-4.5)	0.74
SSTR-TV (mL)	73.2 (17.4-110.7)	111.8 (50.9-235.3)	0.05
TL-SSTR2	272.0 (104.7-568.8)	392.1 (154.3-1589.7)	0.24
MTV (mL)	85.2 (19.4-133.2)	190.8 (73.9-263.4)	0.05
TLG	481.4 (141.7-1084.1)	1088.0 (493.3-2349.2)	0.05

Comparison of the evaluated clinical, IHC-, and imaging-based parameters between patients with M0 and M1 status. The table indicates median (QR) values. Statistical significance of differences between both groups was analyzed in a Mann-Whitney U test.

**Table 2:** Univariate Cox Regression Analysis for OS and TTF

	Median TTF (QR)	HR TTF (95%-CI)	*P* (TTF)	Median OS (QR)	HR OS (95% CI)	*P* (OS)
Sex (female)	7.3 (4.4-15.4)			14.8 (5.7-NE)		
Sex (male)	10.8 (7.9-14.8)	0.77 (0.35-1.67)	0.517	16.1 (10.8-NE)	0.72 (0.30-1.76)	0.484
Age (≤65 y)	8.8 (5.3-12.9)			13.0 (8.5-NE)		
Age (>65 y)	9.7 (5.3-15.4)	0.95 (0.45-1.98)	0.883	16.1 (14.8-NE)	1.41 (0.59-3.36)	0.438
M1	7.3 (5.3-9.5)			10.8 (7.3-NE)		
M0	14.8 (8.1-NA)	0.31 (0.12-0.74)	0.004**	16.2 (14.8-NE)	0.34 (0.12-0.95)	0.040*
High LDH (>418 U/l)	6.9 (1.5-8.8)			10.5 (1.5-16.2)		
Low LDH	10.8 (7.9-16.6)	0.40 (0.18-0.89)	0.024*	16.1 (14.8-NA)	0.42 (0.17-1.03)	0.057
High number of lesions (>7)	7.9 (5.7-9.7)			13.0 (8.6-NE)		
Low number of lesions	14.8 (7.2-NE)	0.42 (0.18-0.94)	0.335	16.2 (14.8-NE)	0.57 (0.22-1.45)	0.239
SSTR2 expression in IHC (score >0)	7.2 (4-8.8)			13 (5.7-NE)		
No SSTR2 expression in IHC	15.4 (9.7-NE)	0.24 (0.09-0.64)	0.004**	16.2 (14.8-NE)	0.26 (0.07-0.83)	0.023*
High hottest lesion SUV_max_ (>27.6)	7.3 (4.4-10.8)			10.8 (5.7-NE)		
Low hottest lesion SUV_max_	12.9 (7.2-15.4)	0.53 (0.23-1.17)	0.115	16.2 (14.8-NE)	0.34 (0.13-0.89)	0.027*
High hottest lesion TLR_peak_ (>2.9)	7.9 (4.4-10.8)			10.8 (7.3-14.8)		
Low hottest lesion TLR_peak_	12.9 (7.2-NE)	0.44 (0.19-0.96)	0.038*	18.1 (16.1-NE)	0.23 (0.07-0.71)	0.011*
High whole-body tumor SUV_mean_ (>5.3)	7.9 (5.3-NE)			13 (8.5-NE)		
Low whole-body tumor SUV_mean_	10.8 (6.2-15.4)	0.6 (0.27-1.32)	0.201	16.2 (10.8-NE)	0.44 (0.16-1.12)	0.084
High whole-body tumor TLR_mean_ (>5.0)	8.8 (0.43-NE)			10.8 (0.43-NE)		
Low whole-body tumor TLR_mean_	9.7 (7.2-14.9)	0.58 (0.21-1.54)	0.297	16.2 (14.8-NE)	0.30 (0.10-0.86)	0.025*
High SSTR-TV (>253ml)	8.8 (5.7-NE)			10.8 (5.7-NE)		
Low SSTR-TV	9.7 (6.6-14.9)	0.76 (0.28-2.05)	0.583	16.2 (14.8-NE)	0.37 (0.21-1.1)	0.068
High TL-SSTR2 (>395)	8.1 (5.2-10.8)			13.0 (8.5-14.8)		
Low TL-SSTR2	12.9 (7.2-16.6)	0.62 (0.28-1.33)	0.218	18.1 (16.1-NE)	0.51 (0.21-1.26)	0.142
High MTV (>264 mL)	9.5 (2.5-10.8)			10.8 (2.5-16.1)		
Low MTV	12.9 (7.3 -15.4)	0.77 (0.32-1.93)	0.581	16.2 (14.8-NE)	0.37 (0.13-0.99)	0.043*
High TLG (>2807)	8.8 (2.5-NE)			10.8 (2.5-NE)		
Low TLG	12.9 (7.5-15.4)	0.37 (0.12-1.07)	0.06	18.1 (14.8-NE)	0.25 (0.08-0.75)	0.010*

Results of Cox-regression analyses of all evaluated clinical, IHC-, and imaging-based parameters for both TTF and OS. The table indicates median TTF and OS for different risk groups, Hazard ratios (HR), and p-values. NE: not estimable, *: p < 0.05, **: p < 0.01.

## Abbreviations

EBUS-TBNA: endobronchial ultrasound-guided transbronchial needle aspiration
GEP-NETs: gastroenteropancreatic neuroendocrine tumors
HR: Hazard ratio
IHC: immunohistochemistry
LDH: serum lactate dehydrogenase
MTV: metabolic tumor volume
NSCLC: non-small cell lung cancer
OS: overall survival
PET: positron emission tomography
PRRT: peptide receptor radionuclide therapy
QR: interquartile range
SCLC: small cell lung cancer
SSO110: satoreotide tetraxetan
SSO120: satoreotide trizoxetan
SSTR-TV: SSTR2-expressing tumor volume
SSTR2: somatostatin receptor type 2
SUV: standardized uptake value
TL-SSTR: total lesion SSTR2-expression
TLG: total lesion glycolysis
TLR: tumor-to-liver ratio
TTF: time to treatment failure
VALG: Veterans Administration Lung Study Group
WHO: World Health Organization

## References

[1] Arnold C (2022). Theranostics could be big business in precision oncology. Nat Med.

[2] Bodei L, Herrmann K, Schöder H, Scott AM, Lewis JS (2022). Radiotheranostics in oncology: current challenges and emerging opportunities. Nat Rev Clin Oncol.

[3] Basu S, Kwee TC, Gatenby R, Saboury B, Torigian DA, Alavi A (2011). Evolving role of molecular imaging with PET in detecting and characterizing heterogeneity of cancer tissue at the primary and metastatic sites, a plausible explanation for failed attempts to cure malignant disorders. Eur J Nucl Med Mol Imaging.

[4] Fischer BM, Mortensen J, Langer SW, Loft A, Berthelsen AK, Petersen BI (2007). A prospective study of PET/CT in initial staging of small-cell lung cancer: comparison with CT, bone scintigraphy and bone marrow analysis. Ann Oncol.

[5] Fischer B, Lassen U, Mortensen J, Larsen S, Loft A, Bertelsen A (2009). Preoperative staging of lung cancer with combined PET-CT. N Engl J Med.

[6] Herbst RS, Heymach JV, Lippman SM (2008). Lung cancer. N Engl J Med.

[7] Gazdar AF, Bunn PA, Minna JD (2017). Small-cell lung cancer: what we know, what we need to know and the path forward. Nat Rev Cancer.

[8] Callison JC Jr, Walker RC, Massion PP (2011). Somatostatin Receptors in Lung Cancer: From Function to Molecular Imaging and Therapeutics. J Lung Cancer.

[9] Sadowski SM, Neychev V, Millo C, Shih J, Nilubol N, Herscovitch P (2016). Prospective Study of 68Ga-DOTATATE Positron Emission Tomography/Computed Tomography for Detecting Gastro-Entero-Pancreatic Neuroendocrine Tumors and Unknown Primary Sites. J Clin Oncol.

[10] Strosberg JR, Caplin ME, Kunz PL, Ruszniewski PB, Bodei L, Hendifar A (2021). (177)Lu-Dotatate plus long-acting octreotide versus highdose long-acting octreotide in patients with midgut neuroendocrine tumours (NETTER-1): final overall survival and long-term safety results from an open-label, randomised, controlled, phase 3 trial. Lancet Oncol.

[11] Kayani I, Conry BG, Groves AM, Win T, Dickson J, Caplin M (2009). A comparison of 68Ga-DOTATATE and 18F-FDG PET/CT in pulmonary neuroendocrine tumors. J Nucl Med.

[12] Naraev BG, Ramirez RA, Kendi AT, Halfdanarson TR (2019). Peptide Receptor Radionuclide Therapy for Patients With Advanced Lung Carcinoids. Clin Lung Cancer.

[13] Lapa C, Hanscheid H, Wild V, Pelzer T, Schirbel A, Werner RA (2016). Somatostatin receptor expression in small cell lung cancer as a prognostic marker and a target for peptide receptor radionuclide therapy. Oncotarget.

[14] Wild D, Fani M, Fischer R, Del Pozzo L, Kaul F, Krebs S (2014). Comparison of somatostatin receptor agonist and antagonist for peptide receptor radionuclide therapy: a pilot study. J Nucl Med.

[15] Nicolas GP, Schreiter N, Kaul F, Uiters J, Bouterfa H, Kaufmann J (2018). Sensitivity Comparison of (68)Ga-OPS202 and (68)Ga-DOTATOC PET/CT in Patients with Gastroenteropancreatic Neuroendocrine Tumors: A Prospective Phase II Imaging Study. J Nucl Med.

[16] Kersting D, Sandach P, Sraieb M, Wiesweg M, Metzenmacher M, Darwiche K (2023). (68)Ga-SSO-120 PET for Initial Staging of Small Cell Lung Cancer Patients: A Single-Center Retrospective Study. J Nucl Med.

[17] Lehman JM, Hoeksema MD, Staub J, Qian J, Harris B, Callison JC (2019). Somatostatin receptor 2 signaling promotes growth and tumor survival in small-cell lung cancer. Int J Cancer.

[18] Weber M, Jentzen W, Hofferber R, Herrmann K, Fendler WP, Conti M (2021). Evaluation of [(68)Ga]Ga-PSMA PET/CT images acquired with a reduced scan time duration in prostate cancer patients using the digital biograph vision. EJNMMI Res.

[19] Wahl RL, Jacene H, Kasamon Y, Lodge MA (2009). From RECIST to PERCIST: Evolving Considerations for PET response criteria in solid tumors. J Nucl Med.

[20] Boellaard R, Delgado-Bolton R, Oyen WJ, Giammarile F, Tatsch K, Eschner W (2015). FDG PET/CT: EANM procedure guidelines for tumour imaging: version 2.0. Eur J Nucl Med Mol Imaging.

[21] Hofheinz F, Butof R, Apostolova I, Zophel K, Steffen IG, Amthauer H (2016). An investigation of the relation between tumor-to-liver ratio (TLR) and tumor-to-blood standard uptake ratio (SUR) in oncological FDG PET. EJNMMI Res.

[22] Choi EK, Park M, Im JJ, Chung Y-A, Oh JK (2020). Prognostic value of 18F-FDG PET/CT metabolic parameters in small cell lung cancer. Journal of International Medical Research.

[23] Denz R, Klaassen-Mielke R, Timmesfeld N (2023). A comparison of different methods to adjust survival curves for confounders. Stat Med.

[24] Nicholson AG, Chansky K, Crowley J, Beyruti R, Kubota K, Turrisi A (2016). The International Association for the Study of Lung Cancer Lung Cancer Staging Project: Proposals for the Revision of the Clinical and Pathologic Staging of Small Cell Lung Cancer in the Forthcoming Eighth Edition of the TNM Classification for Lung Cancer. J Thorac Oncol.

[25] Dingemans AC, Fruh M, Ardizzoni A, Besse B, Faivre-Finn C, Hendriks LE (2021). Small-cell lung cancer: ESMO Clinical Practice Guidelines for diagnosis, treatment and follow-up. Ann Oncol.

[26] Fan S, Zheng H, Zhan Y, Luo J, Zang H, Wang H (2024). Somatostatin receptor2 (SSTR2) expression, prognostic implications, modifications and potential therapeutic strategies associates with head and neck squamous cell carcinomas. Crit Rev Oncol Hematol.

[27] Şen F, Sheikh GT, von Hinten J, Schindele A, Kircher M, Dierks A (2023). In-Vivo Somatostatin-Receptor Expression in Small Cell Lung Cancer as a Prognostic Image Biomarker and Therapeutic Target. Cancers.

[28] Roden AC, Rakshit S, Johnson GB, Jenkins SM, Mansfield AS (2022). Correlation of Somatostatin Receptor 2 Expression, 68Ga-DOTATATE PET Scan and Octreotide Treatment in Thymic Epithelial Tumors. Front Oncol.

[29] He JH, Wang J, Yang YZ, Chen QX, Liu LL, Sun L (2021). SSTR2 is a prognostic factor and a promising therapeutic target in glioma. Am J Transl Res.

[30] Zhang JY, Kunz PL (2022). Making Sense of a Complex Disease: A Practical Approach to Managing Neuroendocrine Tumors. JCO Oncol Pract.

[31] Hope TA, Bergsland EK, Bozkurt MF, Graham M, Heaney AP, Herrmann K (2018). Appropriate Use Criteria for Somatostatin Receptor PET Imaging in Neuroendocrine Tumors. J Nucl Med.

[32] Santo A, Pilotto S, Galetta D, Grossi F, Fasola G, Romano G (2019). Maintenance with lanreotide in small-cell lung cancer expressing somatostatine receptors: A multicenter, randomized, phase 3 trial. Lung Cancer.

[33] Tartarone A, Lerose R, Aieta M (2016). Somatostatin Analog Therapy in Small Cell Lung Cancer. Semin Nucl Med.

[34] Zarogoulidis K, Eleftheriadou E, Kontakiotis T, Gerasimou G, Zarogoulidis P, Sapardanis I (2012). Long acting somatostatin analogues in combination to antineoplastic agents in the treatment of small cell lung cancer patients. Lung Cancer.

[35] Sollini M, Farioli D, Froio A, Chella A, Asti M, Boni R (2013). Brief report on the use of radiolabeled somatostatin analogs for the diagnosis and treatment of metastatic small-cell lung cancer patients. J Thorac Oncol.

[36] Virgolini I, Britton K, Buscombe J, Moncayo R, Paganelli G, Riva P (2002). In- and Y-DOTA-lanreotide: results and implications of the MAURITIUS trial. Semin Nucl Med.

[37] Pless M, Waldherr C, Maecke H, Buitrago C, Herrmann R, Mueller-Brand J (2004). Targeted radiotherapy for small cell lung cancer using 90Yttrium-DOTATOC, an Yttrium-labelled somatostatin analogue: a pilot trial. Lung Cancer.

[38] Kim C, Liu SV, Subramaniam DS, Torres T, Loda M, Esposito G (2020). Phase I study of the (177)Lu-DOTA(0)-Tyr(3)-Octreotate (lutathera) in combination with nivolumab in patients with neuroendocrine tumors of the lung. J Immunother Cancer.

[39] Han G, Hwang E, Lin F, Clift R, Kim D, Guest M (2023). RYZ101 (Ac-225 DOTATATE) Opportunity beyond Gastroenteropancreatic Neuroendocrine Tumors: Preclinical Efficacy in Small-Cell Lung Cancer. Mol Cancer Ther.

[40] Fani M, Nicolas GP, Wild D (2017). Somatostatin Receptor Antagonists for Imaging and Therapy. J Nucl Med.

[41] Krenning EP, Bakker WH, Breeman WA, Koper JW, Kooij PP, Ausema L (1989). Localisation of endocrine-related tumours with radioiodinated analogue of somatostatin. Lancet.

[42] Wild D, Gronbaek H, Navalkissoor S, Haug A, Nicolas GP, Pais B (2023). A phase I/II study of the safety and efficacy of [(177)Lu]Lu-satoreotide tetraxetan in advanced somatostatin receptor-positive neuroendocrine tumours. Eur J Nucl Med Mol Imaging.

[43] Reidy-Lagunes D, Pandit-Taskar N, O'Donoghue JA, Krebs S, Staton KD, Lyashchenko SK (2019). Phase I Trial of Well-Differentiated Neuroendocrine Tumors (NETs) with Radiolabeled Somatostatin Antagonist (177)Lu-Satoreotide Tetraxetan. Clin Cancer Res.

[44] Eigler C, McDougall L, Bauman A, Bernhardt P, Hentschel M, Blackham KA (2024). Radiolabeled Somatostatin Receptor Antagonist Versus Agonist for Peptide Receptor Radionuclide Therapy in Patients with Therapy-Resistant Meningioma: PROMENADE Phase 0 Study. Journal of Nuclear Medicine.

