# Supplementary material for: Theranostics with somatostatin receptor antagonists in SCLC: Correlation of 68Ga-SSO120 PET with immunohistochemistry and survival
Source: Theranostics. 2024 Aug 26;14(14):5400–12. doi: 10.7150/thno.98819 (PMC11413793; doi:10.7150/thno.98819)
Supplement: Supplementary file 1 — Supplementary figures and tables. [file thnov14p5400s1.pdf]

SUPPLEMENTAL MATERIAL to

Theranostics with somatostatin receptor antagonists in SCLC: Correlation of ^{68}Ga-SSO120 PET with immunohistochemistry and survival

Ilektra Antonia Mavroeidi[1,2,3], Anna Romanowicz[2,4], Tristan Haake[5], Johannes Wienker[6,7], Martin Metzenmacher[1,2,7], Kaid Darwiche[6,7], Filiz Oezkan[6,7], Servet Bölükbas[8,9], Martin Stuschke[2,8,10], Lale Umutlu[2,11], Marcel Opitz[2,11], Michael Nader[2,4], Rainer Hamacher[1,2], Jens Siveke[1,2,3], Jane Winantea[6,7], Wolfgang P. Fendler[2,4], Marcel Wiesweg[1,2,7], Wilfried E. E. Eberhardt[1,2,7], Ken Herrmann[2,4,8], Dirk Theegarten[5], Martin Schuler[1,2,7,8], Hubertus Hautzel[2,4], David Kersting[2,4]

[1] Department of Medical Oncology, West German Cancer Center (WTZ), University Hospital Essen, University of Duisburg-Essen, Essen, Germany

[2] German Cancer Consortium (DKTK), Partner Site University Hospital Essen, Essen, Germany

[3] Bridge Institute of Experimental Tumor Therapy (BIT) and Division of Solid Tumor Translational Oncology (DKTK), West German Cancer Center, University Hospital Essen, University of Duisburg-Essen, Essen, Germany.

[4] Department of Nuclear Medicine, West German Cancer Center (WTZ), University Hospital Essen, University of Duisburg-Essen, Essen, Germany

[5] Institute of Pathology, University Hospital Essen, University of Duisburg-Essen, Essen, Germany

[6] Department of Pulmonary Medicine, Section of Interventional Pulmonology, West German Cancer Center (WTZ), University Medicine Essen - Ruhrlandklinik, University of Duisburg-Essen, Essen, Germany

[7] Division of Thoracic Oncology, West German Lung Center, University Medicine Essen - Ruhrlandklinik, University of Duisburg-Essen, Essen, Germany.

[8] National Center for Tumor Diseases (NCT) West, Essen, Germany

[9] Department of Thoracic Surgery and Thoracic Endoscopy, West German Cancer Center (WTZ), University Medicine Essen - Ruhrlandklinik, University of Duisburg-Essen, Essen, Germany

[10] Department of Radiotherapy, West German Cancer Center (WTZ), University Hospital Essen, University of Duisburg-Essen, Essen, Germany

[11] Institute of Diagnostic, Interventional Radiology and Neuroradiology, West German Cancer Center (WTZ), University Hospital Essen, University of Duisburg-Essen, Essen, Germany

Supplemental Table S1: Patient Characteristics

**Age (y)**	
median (range)	65 (50-81)
**Sex (n)**	
male (%)	24 (44.4)
female (%)	30 (55.6)
**T (n)**	
1 (%)	7 (13.0)
2 (%)	6 (11.1)
3 (%)	8 (14.8)
4 (%)	33 (61.1)
**N (n)**	
0 (%)	4 (7.4)
1 (%)	2 (3.7)
2 (%)	20 (37.0)
3 (%)	28 (51.9)
**M (n)**	
0 (%)	21 (38.9)
1 (%)	33 (61.1)
1a (%)	5 (9.2)
1b (%)	7 (13.0)
1c (%)	21 (38.9)

Clinical characteristics of included patients

Supplemental Table S2: Univariate Cox Regression Analysis for OS and TTF (continuous variables)

	HR TTF (95%-CI)	*P* (TTF)	HR OS (95% CI)	*P* (OS)
SSTR2 expression in IHC	0.71 (0.51-0.98)	0.037*	0.61 (0.40-0.93)	0.021*
Hottest lesion SUV_{max}	0.98 (0.96-1.00)	0.047*	0.98 (0.96-1.01)	0.132
Hottest lesion TLR_{peak}	0.94 (0.87-1.02)	0.117	0.91 (0.83-1.00)	0.034*
Whole-body tumor SUV_{mean}	0.97 (0.90-1.05)	0.451	0.96 (0.87-1.05)	0.316
Whole-body tumor TLR_{mean}	0.92 (0.78-1.08)	0.275	0.86 (0.71-1.02)	0.088

Results of Cox-regression analyses of parameters of SSTR2 expression (continuous variables) for both TTF and OS. The table indicates Hazard ratios (HR), and *p*-values.*: $p < 0.05$.

Supplemental Table S3: Comparison of OS and TTF (Stratified Cox analysis)

	HR TTF (95%-CI)	*P* (TTF)	HR OS (95% CI)	*P* (OS)
**SSTR2 expression in IHC (score >0)**				
No SSTR2 expression in IHC	0.32 (0.12-0.87)	0.0244 *	0.32 (0.09-1.09	0.0684
**High hottest lesion SUV_{max} (>27.6)**				
Low hottest lesion SUV_{max}	0.75 (0.33-1.72)	0.499	0.45 (0.16-1.21)	0.113
**High hottest lesion TLR_{peak} (>2.9)**				
Low hottest lesion TLR_{peak}	0.63 (0.28-1.41)	0.259	0.30 (0.09 0.97)	0.0431*
**High whole-body tumor TLR_{mean} (>5.3)**				
Low whole-body tumor TLR_{mean}	0.90 (0.32-2.50)	0.833	0.46 (0.15-1.38)	0.167
**High MTV (>264 mL)**				
Low MTV	0.75 (0.29-1.90)	0.539	0.39 (0.13-1.09)	0.0714
**High TLG (>2807)**				
Low TLG	0.48 (0.16-1.42)	0.183	0.29 (0.09-0.92)	0.034*
**LDH (>418 U/l)**	0.52 (0.22-1.2)	0.1	0.57(*0.22-1.42)	0.2

Results of Stratified Cox regression analyses of clinical, IHC-, and imaging-based parameters for both TTF and OS. The table indicates hazard ratios (HR) for different risk groups for TTF and OS and *p*-values,*: $p < 0.05$, **: $p < 0.01$.

Supplemental Figure S1: ^{68}Ga-SSO120 PET versus ^{18}F-FDG PET

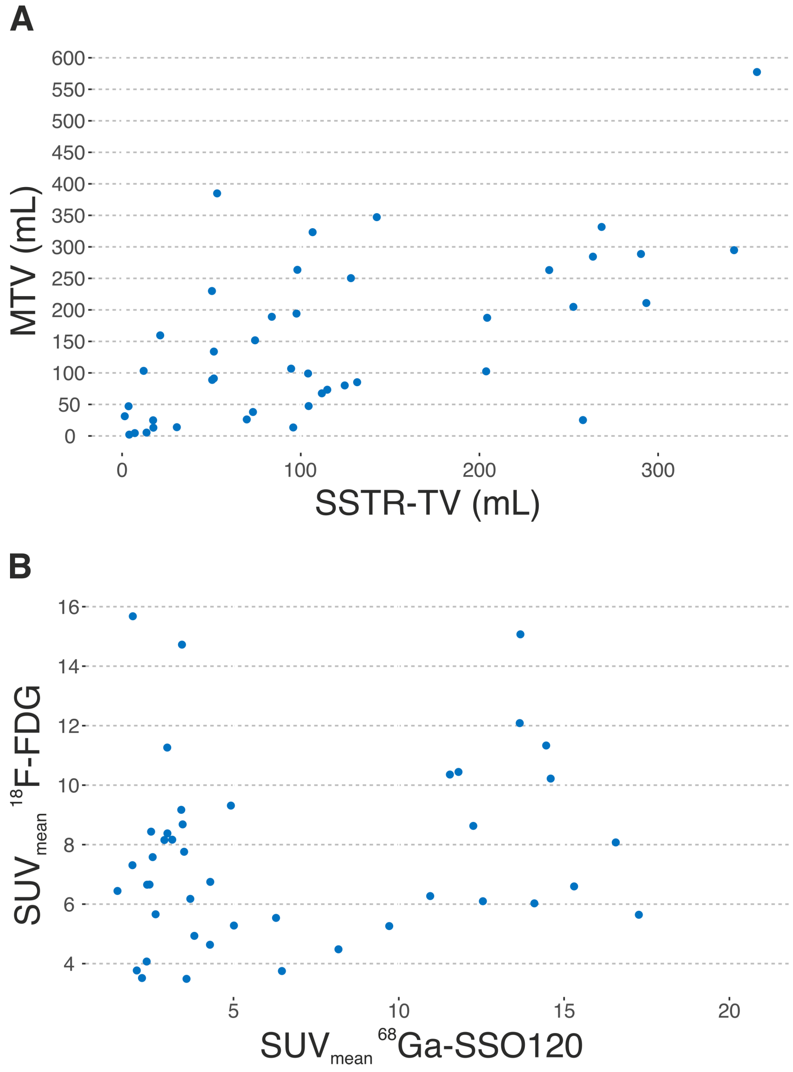

(A) Scatter plot comparing SSTR2-TV and MTV. (B) Scatter plot comparing whole-body tumor SUV_{mean} from ^{68}Ga-SSO120 PET with ^{18}F-FDG PET.

Supplemental Figure S2: Relationship between SUV_{max}/TLR_{peak} and SSTR2 score

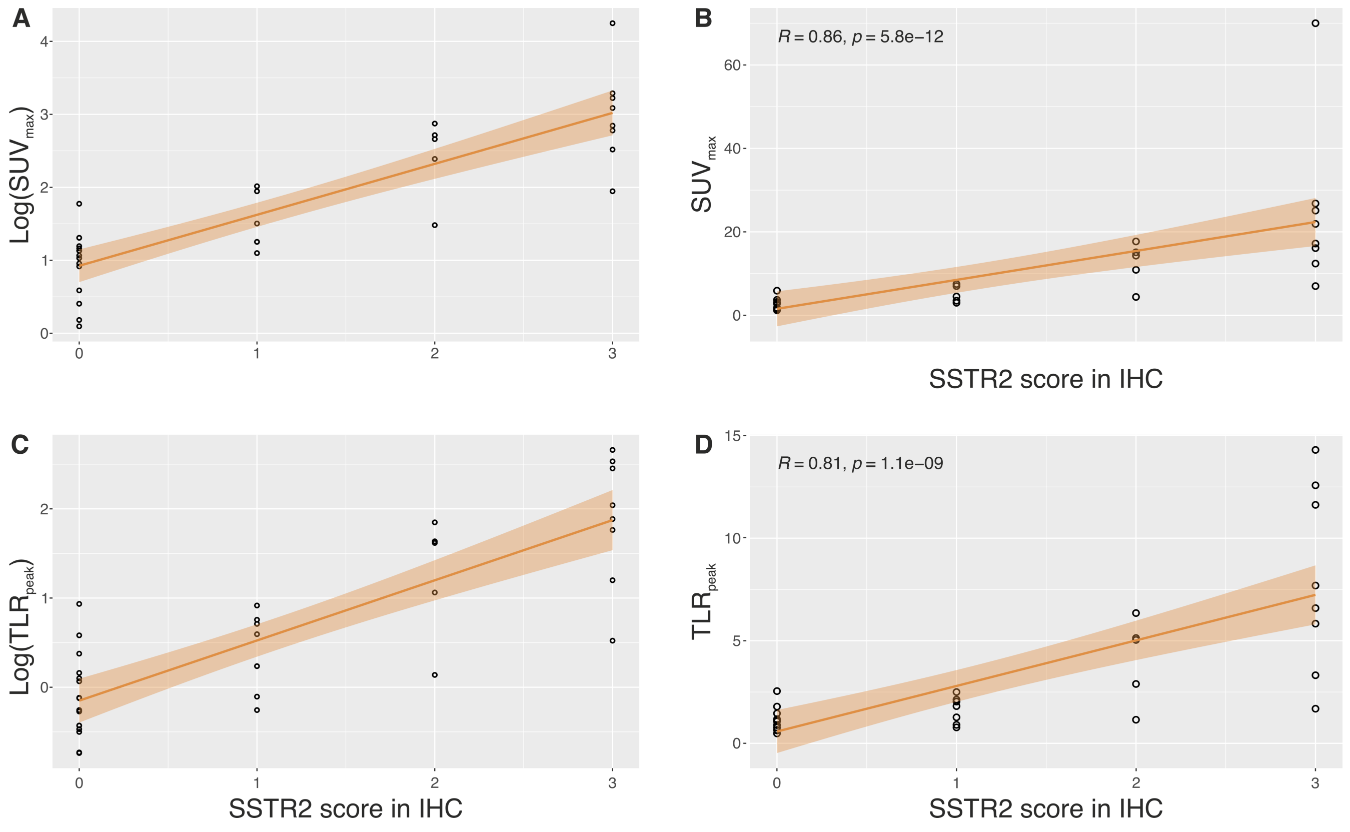

(A) Correlation of log-transformed SUV_{max} with SSTR2 score in IHC. (B) Spearman correlation analysis of SUV_{max} and SSTR2 score in IHC. (C) Correlation of log-transformed TLR_{peak} with SSTR2 score in IHC. (D) Spearman correlation analysis of TLR_{peak} and SSTR2 score in IHC.

Supplemental Figure S3: Survival analyses for TTF

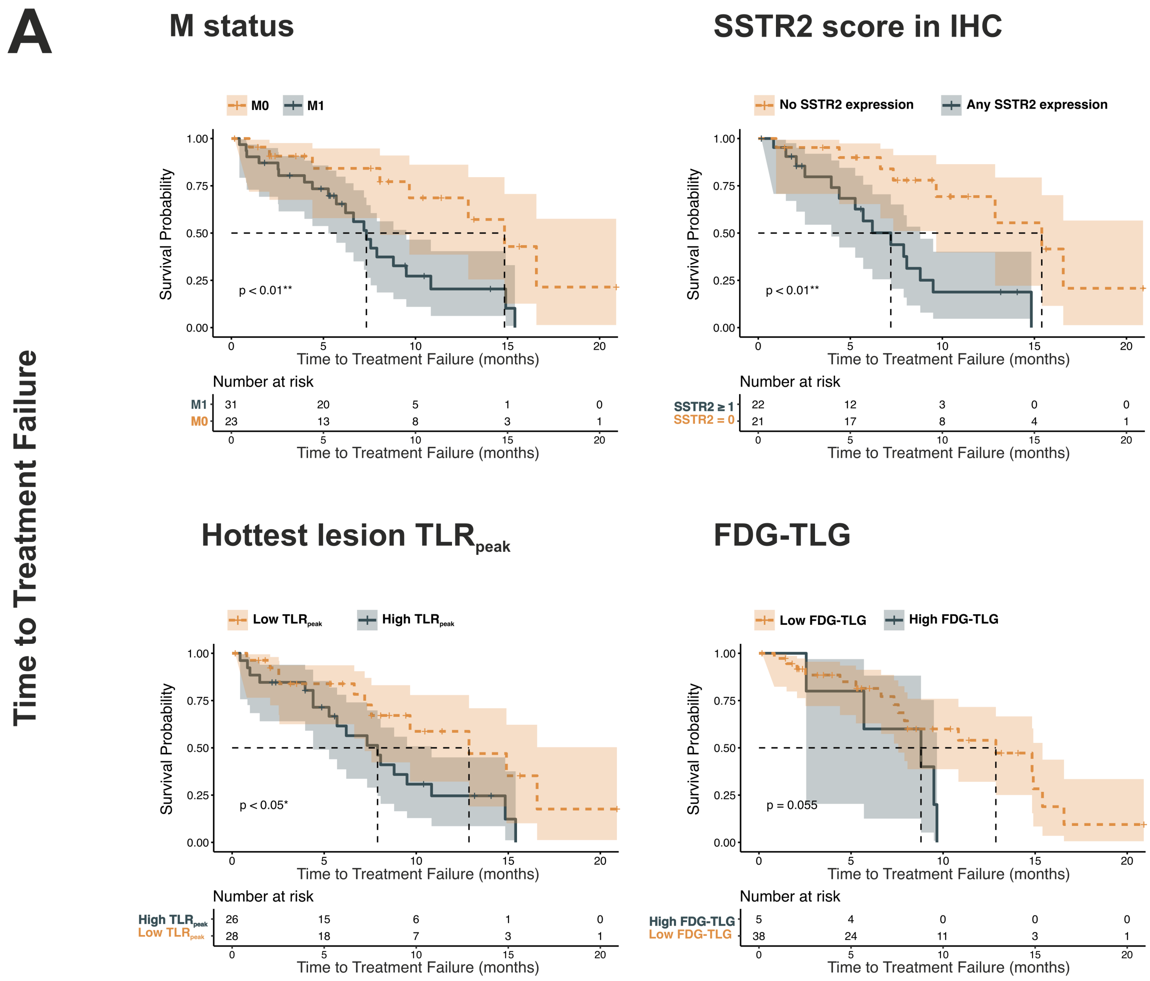

B — Time to Treatment Failure

Variable		N	Hazard ratio		p
**Sex**	f	30		Reference	
	m	24		0.67 (0.30, 1.52)	0.34
**Age**	> 65y	24		Reference	
	≤ 65y	30		1.09 (0.50, 2.37)	0.82
**M status**	M1	31		Reference	
	M0	23		0.50 (0.19, 1.37)	0.18
**LDH**	High	10		Reference	
	Low	44		0.41 (0.17, 0.99)	0.05
**Hottest lesion TLR_{peak}**	High	26		Reference	
	Low	28		0.47 (0.20, 1.09)	0.08

(Hazard ratio axis: 0.2, 0.5, 1, 2)

(A): Kaplan-Meier curves illustrating TTF in correlation to M status, SSTR2 score in IHC, hottest lesion TLR_{peak}, and TLG. (B): Forest plot showing the results of the multivariate Cox regression for

TTF (sex, age (stratified by median), M status, LDH, hottest lesion TLR_{peak}). (High LDH: >418 U/l, high hottest lesion TLR_{peak}: >2.9).

Supplemental Figure S4: Forest Plots SSTR2 Score in IHC

A — Overall Survival

Variable		N	Hazard ratio		p
**Sex**	f	23		Reference	
	m	20		0.61 (0.19, 1.92)	0.40
**Age**	> 65y	19		Reference	
	≤ 65y	24		1.34 (0.49, 3.62)	0.57
**M status**	M1	22		Reference	
	M0	21		0.62 (0.17, 2.28)	0.47
**LDH**	High	8		Reference	
	Low	35		0.29 (0.10, 0.87)	0.03
**SSTR2 score in IHC**	High	22		Reference	
	Low	21		0.22 (0.06, 0.84)	0.03

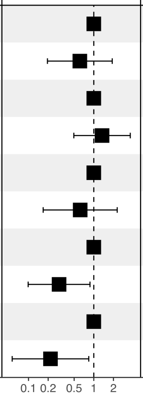

B — Time to Treatment Failure

Variable		N	Hazard ratio		p
**Sex**	f	23		Reference	
	m	20		0.62 (0.23, 1.65)	0.34
**Age**	> 65y	19		Reference	
	≤ 65y	24		0.99 (0.42, 2.34)	0.98
**M status**	M1	22		Reference	
	M0	21		0.64 (0.21, 1.96)	0.43
**LDH**	High	8		Reference	
	Low	35		0.35 (0.13, 0.94)	0.04
**SSTR2 score in IHC**	High	22		Reference	
	Low	21		0.24 (0.08, 0.71)	0.01

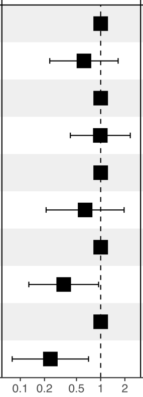

Forest plots showing the results of the multivariate Cox regression for (A) OS and (B) TTF (sex, age (stratified by median), M status, LDH, SSTR2 score in IHC). (High LDH: >418 U/l, high SSTR2 score in IHC: >0).

Supplemental Figure S5: Adjusted Kaplan Meier curves

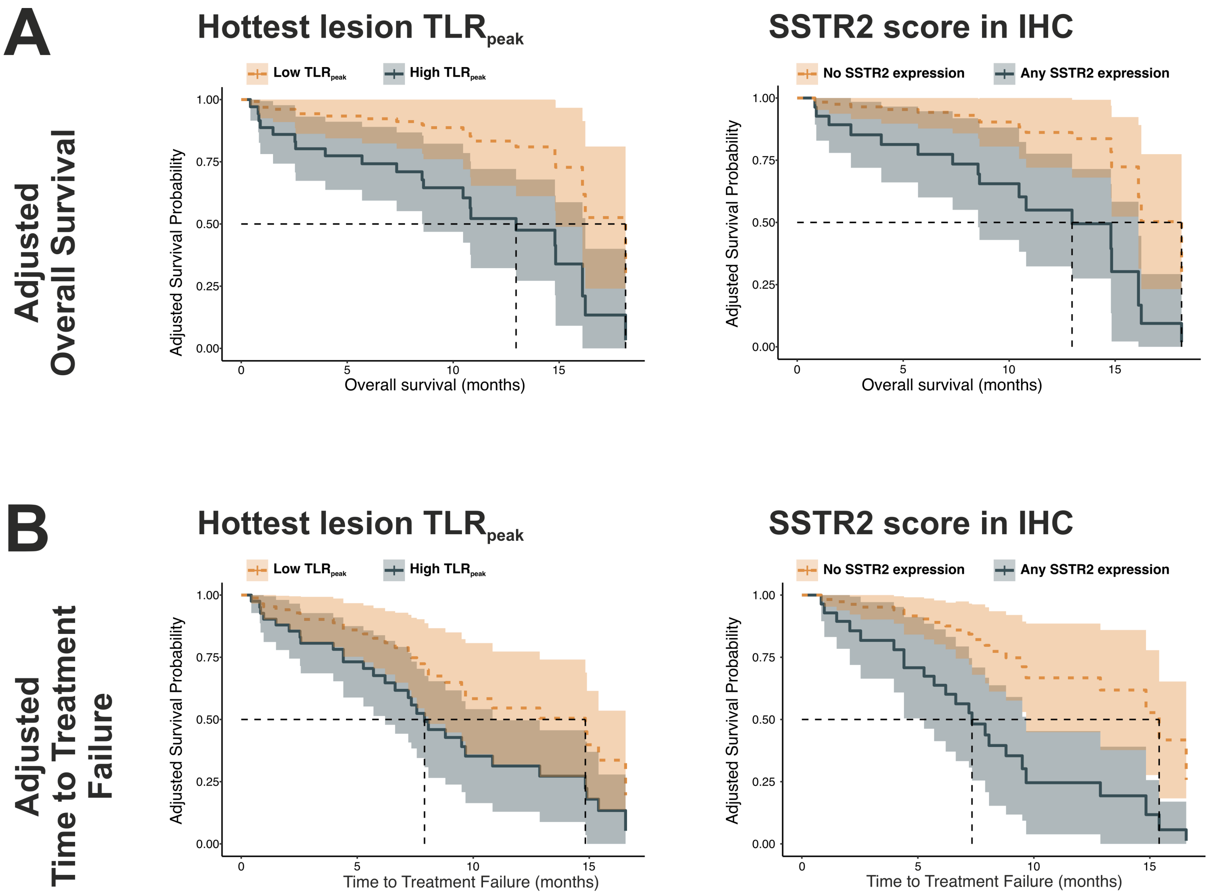

Adjusted Kaplan-Meier curves for hottest lesion TLR_{peak} and SSTR2 score in IHC with (A) OS and (B) TTF.
